# Severe drug-induced liver injury from acipimox and telmisartan co-administration: A case report and review of literature

**DOI:** 10.1097/MD.0000000000044184

**Published:** 2025-08-29

**Authors:** Dan Liu, Shu Liu

**Affiliations:** a Department of Geriatrics, The First Affiliated Hospital of China Medical University, Shenyang City, Liaoning Province, China.

**Keywords:** acipimox, adverse drug reactions, case report, drug-induced liver injury, telmisartan

## Abstract

**Rationale:**

Drug-induced liver injury (DILI) is a prevalent and critical etiology of acute liver failure that often results in substantial health complications and mortality. Acipimox is a pharmacological agent used for lipid reduction, whereas telmisartan is prescribed for hypertension management. We report a case of severe DILI attributed to the concomitant use of acipimox and telmisartan, and we analyze the potential underlying mechanisms.

**Patient concerns:**

A 58-year-old female was admitted to our hospital with anorexia lasting >6 months. The patient had a history of taking oral antihypertensive medication (telmisartan) and lipid-lowering medications (atorvastatin and acipimox), with suspected DILI.

**Diagnoses:**

The patient was diagnosed with DILI.

**Interventions:**

After discontinuation of the suspected hepatotoxic agents telmisartan and acipimox, the patient received anti-inflammatory therapy with glucocorticoids along with hepatoprotective treatment including glutathione, S-adenosylmethionine, and bicyclol.

**Outcomes:**

The patient’s liver function has substantially normalized.

**Lessons:**

This study is the first to highlight a significant case of severe liver injury resulting from concurrent administration of acipimox and telmisartan, which is potentially influenced by factors such as age, sex, drug interactions, and genetic polymorphisms. This study aimed to emphasize the significance of implementing precise and individualized treatment strategies for managing drug combinations for patients with multiple comorbidities.

## 1. Introduction

Drug-induced liver injury (DILI) is a common adverse drug reaction. DILI can result in severe hepatic damage, fulminant hepatitis, or acute liver failure, all of which require immediate medical intervention. In China, the incidence of DILI is markedly higher than that in Western countries, with a reported rate of at least 23.80 cases per 100,000 individuals annually.^[1]^ DILI has become a significant global health concern. In Eastern regions, herbal and dietary supplements have been identified as the predominant contributors to DILI.^[2]^ The existing literature delineates a range of genetic and non-genetic risk factors associated with DILI; however, none have been conclusively validated. It is posited that certain risk factors may modulate susceptibility to DILI in a drug-specific manner. Acipimox, a synthetic derivative of niacin, serves as an effective lipid-lowering agent by inhibiting the release of free fatty acids from adipose tissue.^[3]^ Acipimox demonstrates therapeutic benefits, and its package insert does not indicate any association with abnormal liver function, suggesting a favorable safety profile. Similarly, telmisartan, a modern antihypertensive agent, operates as a conventional angiotensin II type 1 receptor blocker and is extensively employed in the management of hypertension and heart failure. Furthermore, telmisartan’s interaction with peroxisome proliferator-activated receptors (PPARs) may contribute to the mitigation of conditions such as nonalcoholic steatohepatitis and metabolic syndrome, underscoring its diverse pharmacological profile.^[4,5]^ Additionally, the incidence of liver injury associated with telmisartan is rare, enhancing its reputation for safety in clinical practice. Hypertension and dyslipidemia are significant risk factors for cardiovascular and cerebrovascular diseases and frequently coexist in patients, necessitating comprehensive management strategies. As a result, the concurrent administration of antihypertensive and lipid-lowering medications is commonly observed in clinical practice, as healthcare providers endeavor to manage these interrelated health concerns. Nonetheless, the risk assessment associated with the simultaneous use of these medications across diverse populations remains an area in need of substantial enhancement. It is crucial to deepen the comprehension of potential risks linked to combined drug therapies to reduce the incidence of DILI and enhance the overall safety of pharmacological treatments. This report presents a severe case of DILI resulting from the concurrent use of acipimox and telmisartan. Furthermore, it reviews the existing literature on this subject to offer a valuable reference and cautionary guidance for clinicians in developing drug treatment plans for their patients.

## 2. Case presentation

### 2.1. Chief complaints

In March 2024, a 58-year-old female patient was admitted with a primary complaint of anorexia lasting over 6 months.

### 2.2. History of present illness

The patient’s medical history is significant for 13 years of hypertension, with a peak blood pressure reading of 150/110 mm Hg, effectively managed with a daily regimen of 40mg telmisartan, which currently maintains her blood pressure within the desired range. A recent physical examination revealed hyperlipidemia. The patient’s symptoms began after 3 months of oral atorvastatin therapy at a dose of 10 mg once daily. Administration of this medication is associated with a reduction in appetite and intermittent abdominal discomfort. Liver function tests revealed elevated liver enzyme levels, specifically mild increases in alanine aminotransferase (ALT) and aspartate aminotransferase (AST), which led to the discontinuation of atorvastatin and the commencement of hepatoprotective treatment. After 2 weeks of treatment with bicyclol tablets (25 mg orally 3 times a day) and ursodeoxycholic acid capsules (10mg/kg/day), there was an improvement in liver enzyme levels. One month later, with the continuation of hepatoprotective therapy, liver enzyme levels normalized. However, 3 months prior, the patient’s lipid levels experienced an elevation, necessitating the initiation of acipimox therapy (1 capsule orally twice daily) for lipid management. Following 1 month of treatment, there was a subsequent rise in liver enzyme levels, resulting in the cessation of the medication. Presently, the patient reports persistent symptoms of anorexia, shallow sleep, and dark-colored urine, with no notable changes in weight.

### 2.3. History of past illness

Seven-year history of diabetes mellitus, with the highest recorded fasting blood glucose level being 10 mmol/L. Glycemic control is maintained with a regimen of 16 IU of glargine insulin administered once daily and 4 IU of aspart insulin administered 3 times daily, resulting in acceptable fasting blood glucose levels. The patient reports no history of coronary artery disease, hepatitis, or tuberculosis. There has been no recent use of traditional Chinese medicine, and the patient has no other medication history aside from the prescribed antihypertensive, lipid-lowering, and antidiabetic agents.

### 2.4. Physical examination

Upon admission, the physical examination indicated a body temperature of 36.3°C, a blood pressure of 134/78 mm Hg, a heart rate of 77 beats per minute, and a respiratory rate of 16 breaths per minute. The patient was alert and exhibited no conjunctival pallor, jaundice, or cutaneous/mucosal hemorrhages. Lung auscultation revealed clear breath sounds without abnormalities, and the cardiovascular examination was unremarkable. Abdominal examination demonstrated a flat abdomen with no hepatosplenomegaly, tenderness, masses, or rebound tenderness, and there was no evidence of lower limb edema.

### 2.5. Laboratory examinations

Laboratory investigations indicated normal levels of white blood cells, neutrophil percentage, hemoglobin, and platelet count. Additionally, C-reactive protein, coagulation profile, D-dimer, myocardial enzymes, and troponin levels were all within normal limits. Hepatitis virus screening was negative. The liver function tests revealed elevated levels of AST at 590 U/L (N < 35), ALT at 629 U/L (N < 40), alkaline phosphatase (ALP) at 318 U/L (N < 135), gamma-glutamyl transferase (GGT) at 269 U/L (N < 45), total bilirubin (TBIL) at 75.1 µmol/L (N < 21), and direct bilirubin (DBIL) at 58.4 µmol/L (N < 8). In contrast, serum albumin levels remained within the normal range at 39.8 g/L. The assessment of liver-related autoantibodies was largely negative, except for the presence of a positive antinuclear antibody with a titer of 1:100.

### 2.6. Imaging examinations

**Figure 1.**
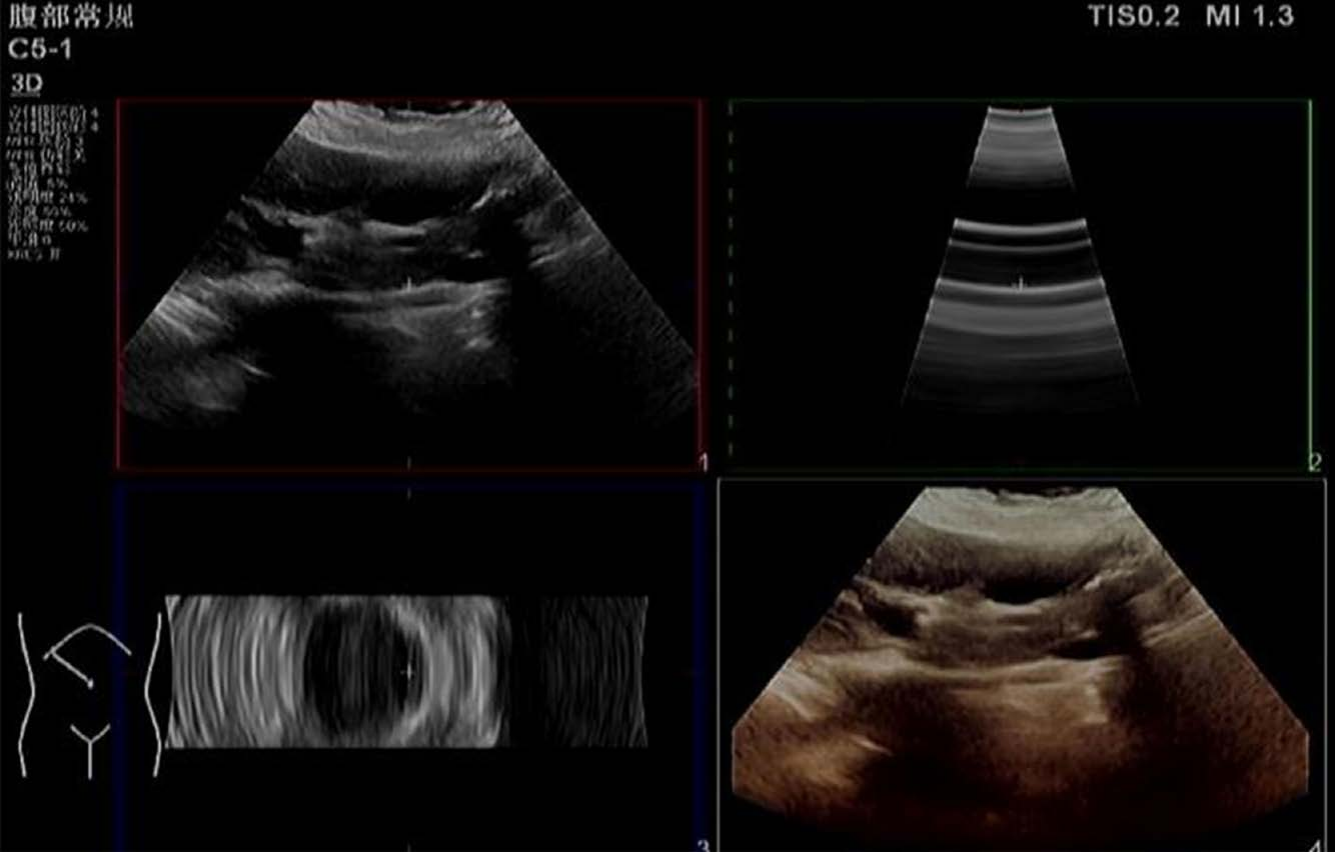
Ultrasound examination of the Liver. Liver ultrasonography revealed that the liver’s morphology and dimensions were within normal limits. However, the liver surface appeared irregular, and the liver edge was either sharp or blunt. The echotexture of the liver parenchyma was coarse. The hepatic vein was clearly visualized, although the vessel wall was irregular. There was no evidence of portal vein system dilation, and the intrahepatic blood flow was well visualized.

An abdominal ultrasound demonstrated coarse liver parenchyma, suggestive of liver damage, along with the presence of liver cysts (Fig. 1).

### 2.7. Further diagnostic work-up

**Figure 2.**
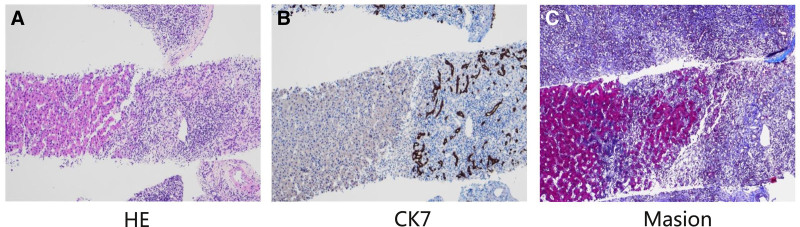
Pathological results of liver biopsy. (a) The HE staining showed that hepatocytes were severely damaged, with obvious fusion necrosis and bridging necrosis, mixed inflammatory cell infiltration, and bile duct hyperplasia, which was consistent with acute severe liver injury. (b) Immunohistochemistry showed CK7 (*Small bile duct*+), CK19 (*Small bile duct*+), CD163 (*Liver Sinusoidal Endothelium*+), CD68 (*histiocytosis+*), CD43 (*vessels*+), HBcAg (−), HBsAg (−), MUM1 (*Plasma cell*-), IgG4 (−). (c) Special stains showed Massion (+), PAS (+), reticulated fiber (+), D-PAS (−), acid-fast staining (−), iron Staining (−), CopperStaining (−). (+) means positive, (−) means negative. CD = cluster of differentiation, CK= cytokeratin, HBcAg = hepatitis B core antigen, HBsAg = hepatitis B surface antigen, HE = hematoxylin–eosin staining, IgG4 = immunoglobulin G class 4, MUM1 = multiple myeloma oncogene 1, PAS = periodic acid-Schiff staining.

Based on the patient’s clinical history and laboratory findings, a Roussel Uclaf Causality Assessment Method (RUCAM) score of 7 was calculated, indicating a probable DILI.^[6]^ Given the persistent elevation in AST levels, we recommended performing a liver biopsy to obtain a definitive diagnosis. The histopathological analysis of the liver biopsy specimen (Fig. 2) revealed substantial hepatocyte destruction, characterized by pronounced fusion and bridging necrosis, accompanied by mixed inflammatory cell infiltration and bile duct proliferation. These findings are indicative of acute severe liver injury, most likely induced by medication.

The immunohistochemical analysis revealed positive markers for CD163, indicative of sinusoidal endothelium; CD68, associated with macrophages; CD34, related to vascular structures; and CK7 and CK19, both of which are markers for small bile ducts. Negative markers included HBcAg, HBsAg, and IgG4, whereas MUM1 was positive, indicating the presence of plasma cells. Molecular pathology findings confirmed the validity of the EBV positive control, although EBV itself tested negative. Results from special staining techniques demonstrated negativity for D-PAS, acid-fast stain, iron stain, and copper stain, while Masson trichrome stain, PAS, and reticular fiber stain yielded positive results.

### 2.8. Final diagnosis

The patient was diagnosed with DILI based on the pathological results.

### 2.9. Treatment

**Figure 3.**
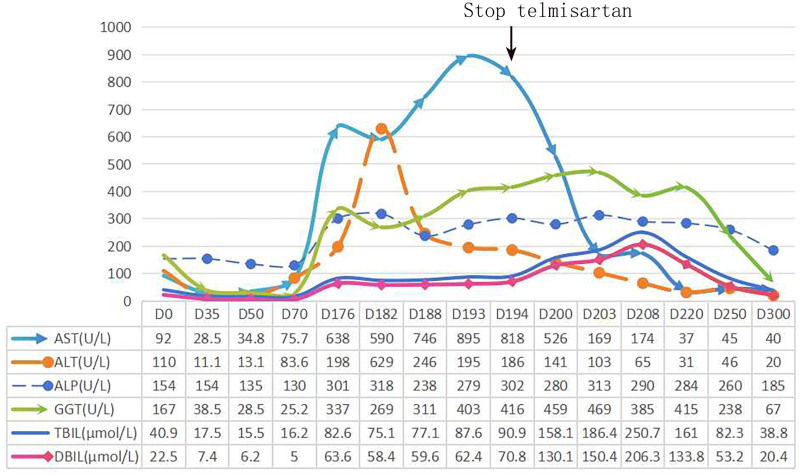
The trend of liver function changes of the patient. ALP = alkaline phosphatase, ALT = alanine aminotransferase, AST = aspartate aminotransferase, DBIL = direct bilirubin, GGT = gamma-glutamyl transferase, TBIL = total bilirubin.

**Figure 4.**
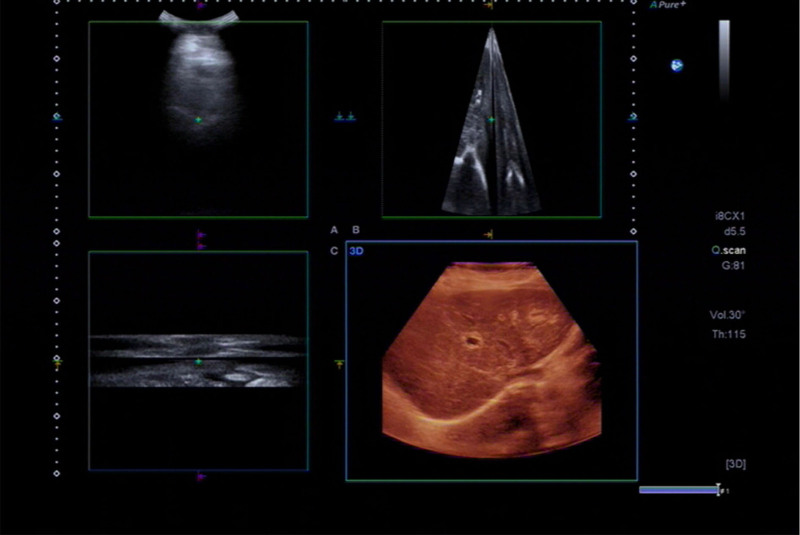
Follow-up liver ultrasonography.

We discontinued all suspected medications; however, the patient opted to continue telmisartan, which she had been using for nearly a decade to manage her chronic hypertension. To address the abnormalities in liver function, we administered ademetionine disulfate sodium (SMT, 1000 mg/d), glutathione (1.2 g/d), and glycyrrhizin(200 mg/d)intravenously. Furthermore, the patient was given oral bicyclol (25 mg orally 3 times a day). After 1 week of treatment, liver function tests indicated the following results: AST at 746 U/L, ALT at 236 U/L, ALP at 238 U/L, GGT at 311 U/L, TBIL at 77.1 µmol/L, and DBIL at 59.6 µmol/L. Following a review of the pathological findings, the patient’s medication regimen was assessed. It was recommended that telmisartan be discontinued and replaced with a calcium channel blocker for hypertension management. After discontinuation of telmisartan, follow-up liver function tests demonstrated a marked reduction in AST levels, whereas bilirubin levels significantly increased (Fig. 3). The specific laboratory values were as follows: AST: 174 U/L, ALT: 65 U/L, ALP: 290 U/L, GGT: 385 U/L, TBIL: 250.7 µmol/L, and DBIL: 206.3 µmol/L. Follow-up liver ultrasonography showed no significant changes compared to the previous examination (Fig. 4). We hypothesized that the elevated bilirubin levels were attributable to an obstruction of the intrahepatic bile duct, likely resulting from extensive hepatocellular damage. Initial treatment with multiple hepatoprotective agents was ineffective, and the patient had no plan for dialysis. Simultaneously, it is important to consider that persistent abnormalities in liver function among patients may be influenced by certain drug-induced immunological factors. Consequently, a short-term trial of corticosteroid therapy was initiated in consultation with the patient. Glucocorticoid therapy was initiated with intravenous methylprednisolone at a dose of 40 mg once daily, resulting in an overall favorable clinical response. After 6 days, liver function tests demonstrated improvement, with the following results: AST at 37 U/L, ALT at 31 U/L, ALP at 284 U/L, GGT at 415 U/L, TBIL at 161.0 µmol/L, and DBIL at 133.8 µmol/L. Upon discharge, the patient gradually tapered the steroid dosage. Nearly 3 months post-discharge, outpatient liver function tests revealed substantial improvement in enzyme and bilirubin levels: AST at 40 U/L, ALT at 20 U/L, ALP at 185 U/L, GGT at 67 U/L, TBIL at 38.8 µmol/L, and DBIL at 20.4 µmol/L.

### 2.10. Outcome and follow-up

**Figure 5.**
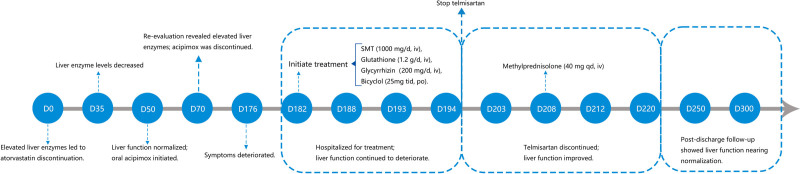
Timeline of the events described. iv = intravenously, po = oral, SMT = ademetionine disulfate sodium, tid = 3 times a day.

The patient adhered well to the prescribed medication regimen. Follow-up evaluations demonstrated normalization of ALT levels and marked improvement in other laboratory parameters (the timeline is shown in Figure 5). The patient expressed satisfaction with the treatment outcomes.

## 3. Discussion

The patient had no prior history of autoimmune disease or viral hepatitis, as all tests for autoimmune-related and viral antibodies yielded negative results. Prior to admission, the patient exhibited elevated ALT and ALP levels, with an *R*-value of 2.05 (*R*-value = [measured ALT/ALT upper limit of normal]/[measured ALP/ALP upper limit of normal]), suggesting mixed liver injury.^[7]^ The RUCAM score for this patient was 7, which, in conjunction with the biopsy results, supports the diagnosis of DILI. An alternative Naranjo assessment yielded a score of 1 for acipimox and 2 for telmisartan, suggesting a greater likelihood that the liver injury resulted from the combined effects of both medications. Based on the patient’s medication history and the observed alterations in liver function following the discontinuation of acipimox and telmisartan, we hypothesized that the acute liver injury was attributable to the concurrent use of these medications. This association has been infrequently documented in the literature and may provide valuable insight for future evaluations of drug interactions.

The liver is integral to the metabolism and biotransformation of most pharmaceuticals. DILI can be categorized according to its mechanism of action into the same 3 types: direct, indirect, and idiosyncratic forms. Certain pharmaceuticals have the potential to cause direct hepatic damage, whereas others may induce liver injury through metabolites produced during hepatic metabolism.^[8,9]^ The process of drug metabolism encompasses a variety of reactions and enzymes, which can lead to the generation of reactive oxygen species that further aggravate hepatocellular damage.^[10]^ Genetic research has elucidated associations between DILI and polymorphisms in drug-metabolizing enzymes, drug transporters, and human leukocyte antigens.^[11–13]^ Consequently, multiple factors can contribute to DILI, with any component influencing drug metabolism, transport, oxidative defense, immune response, or genetic polymorphisms could potentially increase the risk of DILI.

Cases of liver injury associated with telmisartan and acipimox are relatively uncommon when considered separately.^[14,15]^ In this case, the potential causes of severe DILI resulting from the combination of acipimox and telmisartan include the following factors:

1. Basic conditions such as age & gender: Cases of liver injury caused by acipimox are infrequently reported in the medical literature. A review of PUBMED over the past 2 decades identified a case study involving a 51-year-old woman who developed hepatocellular DILI after a 4-week course of oral acipimox. Her liver function returned to normal within 3 months of discontinuing the medication. Notably, both patients in the identified cases were women in their fifties, suggesting that age and gender might be risk factors for DILI.^[16]^ Another preliminary study reported a case of liver injury in a male patient in his 40s after 3 days of acipimox administration.^[17]^ Nonetheless, additional research is warranted. Furthermore, the expression of uridine diphosphate-glucuronosyltransferase (UGT) exhibits gender-specific differences, with men generally presenting lower levels and activity compared to women.^[18]^ This indicates that women may achieve therapeutic effects with reduced dosages and extended administration intervals. Patients with type 2 diabetes, characterized by insulin resistance that disrupts glucose and lipid metabolism, may also be susceptible to hepatic damage.^[19]^ It is plausible that the underlying condition may be a contributing factor to the persistent liver injury observed in this case.
2. Drug interactions: acipimox has the potential to initially elevate liver enzyme levels, which may result in severe DILI and an extended recovery period. This is primarily attributed to its additive effect when used concomitantly with telmisartan. Telmisartan is associated with elevated serum transaminase levels in fewer than 2% of cases, a rate comparable to that observed with placebo treatments in clinical trials. These elevations are typically transient and rarely necessitate dosage modifications.^[20]^ In contrast, the bile salt export pump (BSEP) plays a crucial role in bile acid transport, and telmisartan has been shown to inhibit its function, potentially leading to cholestasis.^[21]^ However, there have been no documented cases of direct hepatotoxicity linked to telmisartan. Telmisartan seems to function as a partial agonist of the PPAR-γ.^[5]^ The interaction of telmisartan with PPARs may ameliorate myocardial ischemia-reperfusion injury in diabetic rat models.^[22]^ Additionally, it inhibits lipogenesis and promotes lipolysis, thereby effectively mitigating weight gain and reducing the risk of obesity.^[23]^ Several studies indicate that telmisartan may alleviate biochemically induced liver damage.^[24–26]^ Furthermore, some research suggests that telmisartan may exert protective effects against nonalcoholic liver fibrosis.^[27]^ Nevertheless, telmisartan is contraindicated in patients with severe hepatic impairment. For individuals with mild to moderate hepatic impairment, the daily dosage should not exceed 40 mg. In this particular case, the patient exhibited elevated liver enzyme levels following 1 month of acipimox treatment while maintaining a daily intake of 40 mg of telmisartan. Although the prescribed dosage was not exceeded, telmisartan is not recommended for use in patients with moderate to severe hepatic impairment. Although the patient had no history of liver injury during long-term antihypertensive treatment with telmisartan, the subsequent development of hepatic injury following combination therapy for dyslipidemia suggests that telmisartan may have contributed to exacerbating liver damage. Interindividual variability in drug concentration could potentially contribute to cholestasis and delayed hepatic recovery.
3. Genetic polymorphisms: telmisartan demonstrates significant interindividual variability in blood concentration levels. This variability may be attributed to polymorphisms in the UGT genes.^[18]^ UGT serves as a crucial enzyme in phase II metabolism, facilitating the clearance of telmisartan from the body by conjugating it to glucuronides, thereby enhancing its solubility and promoting excretion.^[18]^ Research indicates that the mutation frequencies of UGT1A3 in the Chinese population are 26.8% for T31C, 26.8% for G81A, and 10.4% for T104C.^[28]^ In contrast, these mutation frequencies are markedly higher in Caucasian populations, with 65% for T31C, 65% for G81A, and 58% for T104C.^[29]^ A study investigating the association between UGT1A3 gene polymorphisms and the pharmacokinetics of telmisartan in 33 healthy Japanese males identified significant variations in enzyme activity and clearance rates among individuals carrying the UGT1A3*1a/*1a, *2a, and *4a variants.^[30]^
4. Rechallenge: Re-administration of a drug that previously induced liver injury can result in adverse effects.^[31]^ In this case, the patient’s medical history reveals that atorvastatin was discontinued 3 months prior due to elevated liver enzyme levels, which were suspected to be drug-induced. After resuming oral administration of acipimox, the liver enzyme levels increased again within 3 months. Although atorvastatin and acipimox are distinct pharmacological agents, both are classified as lipid-lowering medications. Frequent liver damage over a short period can compromise the liver’s regenerative capacity.

This study has several limitations. Due to the lack of specific diagnostic biomarkers, DILI remains a diagnosis of exclusion. First, causality assessment in this patient was challenging owing to a history of multiple concomitant medications with prolonged exposure. Although acipimox and telmisartan were identified as the primary suspect agents, the potential confounding influence of other medications cannot be entirely ruled out. Second, in the absence of pharmacokinetic or pharmacogenetic analyses, the mechanistic role of potential drug–drug interactions or genetic polymorphisms remains speculative.

However, exclusion of alternative etiologies (viral, autoimmune, alcoholic), a RUCAM causality assessment score of 7, and compatible liver biopsy findings collectively support the DILI diagnosis. Furthermore, the observed improvement in liver function following discontinuation of acipimox and telmisartan reinforces this causal relationship.

It is important to note that acipimox is a derivative of niacin. Niacin (vitamin B3) is commonly used to manage blood lipid levels but can cause hepatotoxicity. Its metabolites may contribute to flushing and liver toxicity,^[32]^ though the exact mechanisms are unclear. Despite this, niacin can protect the liver in some cases, such as preventing experimental liver fibrosis through antioxidant effects and reduced Transforming Growth Factor-Beta expression.^[33]^ It can be inferred that when niacin is co-administered with telmisartan, careful dose management and liver function monitoring are essential to prevent potential toxicity.

## 4. Conclusion

As the global population ages, the United Nations projects that by the mid-21st century, the number of individuals aged 65 and older will more than double.^[34]^ A significant proportion of this demographic manages multiple chronic conditions and consequently takes various medications, thereby elevating the risk of DILI. This case report indicates that while telmisartan and acipimox infrequently cause DILI when administered independently, their concurrent or repeated use may result in hepatic injury. This case underscores the complexities in managing hyperlipidemia in patients with established hypertension and highlights the potential hepatotoxic interactions among commonly prescribed antihypertensive and lipid-lowering agents. Consequently, adherence to drug compatibility guidelines is imperative when administering combination therapies, as this mitigates the risk of DILI resulting from drug interactions. Clinicians must maintain a vigilant approach, thoroughly evaluating patients’ underlying health conditions while aiming for the early prediction, detection, diagnosis, and management of DILI. Future research endeavors should concentrate on a comprehensive and systematic investigation of the mechanisms underlying DILI, thereby yielding critical insights for its prevention, diagnosis, and treatment.

## Author contributions

**Conceptualization:** Dan Liu.

**Data curation:** Dan Liu.

**Investigation:** Dan Liu.

**Supervision:** Shu Liu.

**Validation:** Shu Liu.

**Visualization:** Shu Liu.

**Writing – original draft:** Dan Liu.

**Writing – review & editing:** Shu Liu.
